# Differentiation of *Trichuris* species using a morphometric approach

**DOI:** 10.1016/j.ijppaw.2019.05.012

**Published:** 2019-05-31

**Authors:** A.M. García-Sánchez, J. Rivero, R. Callejón, A. Zurita, M. Reguera-Gomez, M.A. Valero, C. Cutillas

**Affiliations:** a Department of Microbiology and Parasitology, Faculty of Pharmacy, University of Seville, Professor García González 2, 41012, Seville, Spain; b Departamento de Parasitología, Facultad de Farmacia, Universidad de Valencia, Av. Vicent Andrés Estellés s/n, 46100, Burjassot, Valencia, Spain

**Keywords:** Primates, *Trichuris*, specific differentiation, Morphometrics

## Abstract

*Trichuris trichiura* is a nematode considered as the whipworm present in humans and primates. The systematics of the genus *Trichuris* is complex. Morphological studies of *Trichuris* isolated from primates and humans conclude that the species infecting these hosts is the same. Furthermore, numerous molecular studies have been carried out so far to discriminate parasite species from humans and Non-Human Primates using molecular techniques, but these studies were not performed in combination with a parallel morphological study. The hypothesised existence of more species of *Trichuris* in primates opens the possibility to revise the zoonotic potential and host specificity of *T. trichiura* and other putative new species of whipworms.

In the present work, a study of *Trichuris* Roederer, 1761 (Nematoda:Trichuridae) parasitizing *C. g. kikuyensis*, *P. ursinus*, *Macaca sylvanus*, *Pan troglodytes*, and *Sus scrofa domestica* has been carried out using modern morphometric techniques in order to differentiate populations of *Trichuris* isolated from four species of captive NHP from different geographical regions, and swine, respectively.

The results obtained revealed strong support for geometrical morphometrics as a useful tool to differentiate male *Trichuris* populations. Therefore, morphometrics in combination with other techniques, such as molecular biology analyses, ought to be applied to further the differentiation of male populations.

On the other hand, morphometrics applied to female *Trichuris* species does not seem to contribute new information as all the measurements combinations of obtained from females always showed similar results.

## Highlights

- Geometrical morphometrics is an useful tool to differentiate male *Trichuris* populations.
- Geometrical morphometrics results are in agreement with the molecular biology analysis applied to the adult male samples that allow the identification of different species of *Trichuris.*
- The hypothesised existence of different species of *Trichuris* in primates opens the possibility to revise the zoonotic potential and host specificity of *T. trichiura* and other putative new species of whipworms.

## Graphical abstract

**Figure:**
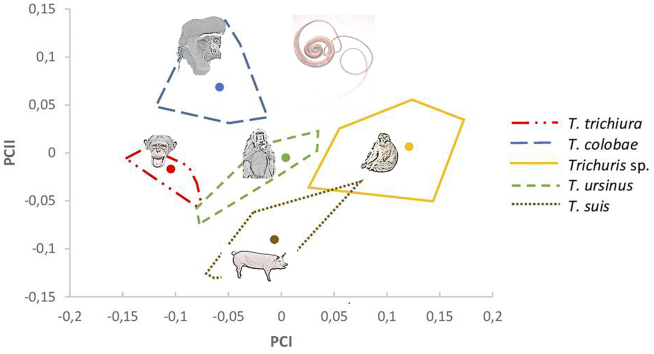

## 1 Introduction

*Trichuris* species are nematodes parasitizing the caecum of different hosts. The specific differentiation of the genus *Trichuris* has been the subject of a long-ranging controversy. Several morphological studies have been reported (see Cutillas et al., 2014), since Dujardin (1845) reviewed the genus for the first time. Chandler (1930) and Knight (1971) found that the spicule length is the most dependable character for *Trichuris* species differentiation. Nevertheless, the spicule length of *Trichuris trichiura* and that of *Trichuris suis* overlaps. Furthermore, Spakulová and Lýsek (1981) found that the male and female of *T*. *suis* from abattoir pigs differed significantly from *T. suis* from wild boars.

Morphometric characters of nematode parasites such as body length, body width, oesophagus length, spicule length, etc can vary according to a variety of environmental factors, including host. Thus, Knight (1984) reported that the morphological characteristics of *Trichuris ovis* were affected by the host to a greater extent than by geographical area.

Several features, such as the presence/absence of the spicule tube, the shape and distribution of the spines of the spicule sheath, length of the spicule and the cloacal tube, the shape of the proximal and distal cloacal tube, and the vulvar morphology, along with classic morphometric characteristics have been found to have a high discriminatory value to differentiate *Trichuris* species (Correa et al., 1992; Suriano and Navone, 1994; Robles et al., 2006, 2014; Callejón et al., 2017). Furthermore, some studies (Kikuchi, 1974a, 1974b; Tenora et al., 1993, 1997; Robles et al., 2006; Cutillas et al., 2009, 2014; Callejón et al., 2017) have used scanning electron microscopy (SEM) as a useful diagnostic tool. *Trichuris* species have been described with a narrow range of anatomic and biometric characteristics, and they have been insufficiently compared with their congeneric species (Robles et al., 2014).

Cutillas et al. (2014) proposed a new species, *Trichuris colobae*, for a Non-Human Primate (NHP), based on different parameters that significantly discriminated *T. suis* and *T*. *trichiura* to *T. colobae* from *Colobus guereza kikuyensis*. Furthermore, Callejón et al. (2017) reported, in a morphological and morphometric study, that *Trichuris ursinus* from another NHP (*Papio ursinus*) differed significantly from *T. trichiura* (nine different characters) and *T. colobae* (six different characters). Also, *T. ursinus* shows features close to *T. suis* (only three different characters). *Trichuris* specimens were measured according to parameters reported by Spakulová and Lýsek (1981), Suriano and Navone (1994) and Robles et al. (2006) who summarized the parameters used in recent years.

Traditional parasitological research on *T. trichiura* from humans and NHPs has focused on differentiating it from *T. suis* found in pigs (Beer, 1976; Ooi et al., 1993; Cutillas et al., 2009; Nissen et al., 2012; Liu et al., 2012). Morphological studies of *Trichuris* isolated from primates and humans conclude that the species infecting these hosts is the same, although slight morphological variations are distinguishable when scanning electron microscopy is used (Ooi et al., 1993). Nevertheless, these studies were based on a few morphological characters such as the total length or spicule length, etc., but not on discriminative analysis of several significant morpho-biometric parameters using statistical tests. Thus, numerous molecular studies have been carried out so far to discriminate parasite species from humans and NHPs using molecular techniques, but these studies were not performed in combination with a parallel morphological study. Ravasi et al. (2012) suggested the need for morphological analysis of *Trichuris* sp. adult worms collected from *P. ursinus* (Chacma baboon) from South Africa to determine whether the genetic lineages correspond to different morphological species. In this sense, Callejón et al. (2017) reported in a morphological and morphometric study that *T. ursinus* from Chacma baboon (*P. ursinus*) differed significantly from *T. trichiura* (nine different characters) and *T. colobae* (six different characters) and showed features close to *T. suis*.

The systematics of the genus *Trichuris* is complex. Although morphological variation has been quantified by modern morphometric techniques in parasite as arthropods (Zurita et al., 2019; Santillán-Guayasamín et al., 2017) and helminths (Ashrafi et al., 2015; Valero et al., 2018), it has never been applied to *Trichuris* species.

Our findings suggest the need for morphometric analyses of adult *Trichuris* species of different NHPs to determine whether the genetic lineages correspond to different species and whether there are significant morphological features and biometric data that can be used to distinguish them from *T. suis* and *T. trichiura*. Thus, in the present work, a study of *Trichuris* Roederer, 1761 (Nematoda: Trichuridae) parasitizing *C. g. kikuyensis*, *P. ursinus*, *Macaca sylvanus*, *Pan troglodytes*, and *Sus scrofa domestica* has been carried out using modern morphometric techniques in order to differentiate populations of *Trichuris* isolated from four species of captive NHP from different geographical regions, and swine, respectively.

## 2 Material and methods

### 2.1 Collection of samples

120 adult *Trichuris* sp. specimens (60 males and 60 females) were collected from the caecum of adult primates (*M. sylvanus*, *P. troglodytes*, *C.g. kikuyensis* and *P. ursinus*) and 30 adults from pigs, thoroughly washed with saline solution of 0.9% sodium chloride, and subsequently stored in 70% ethanol.

30 adults (15 males and 15 females) of *Trichuris* sp. were collected from the caecum of *M. sylvanus* died in the zoo of Castellar (Cádiz, Spain). Genetic analysis are unpublished.

30 adults (15 males and 15 females) of *T. ursinus* were collected from Chacma baboons (*P. ursinus*) from the Cape Peninsula, South Africa. These specimens were obtained through the assistance of colleagues (see Acknowledgements). Genetic data are published (Callejón et al., 2017).

Adults of *T. colobae* were collected from the caecum of a primate (*C. g. kikuyensis*) died in the Zoo of Fuengirola (Málaga, Spain). Genetic data are published (Cutillas et al., 2014).

Adults of *T. suis* were collected from the caecum of swine (*S. s. domestica*) slaughtered at abattoirs in different locations in the provinces of Seville and Huelva (Spain). Genetic data are published (Cutillas et al., 2009).

Adults of *T. trichiura* were collected from the caecum of chimpanzees died in the zoo in Barcelona (Spain). Genetic data are published (Cutillas et al., 2009).

### 2.2 Morphological studies and metric data processing

Morphobiometric data of *T. suis* and *T. trichiura* from the chimpanzee were those cited by Cutillas et al. (2009). The identification of these species was carried out in accordance with previous studies (Oliveros et al., 2000; Cutillas et al., 2002, 2014, 2004; 2007; Callejón et al., 2017).

The collected specimens of *Trichuris* sp. were measured. The measurements considered in this work are the most representative reported by Spakulová and Lysek (1981), Suriano and Navone (1994) and Robles et al. (2006). In addition, a comparative study of morpho-biometric data of five *Trichuris* species was performed. Descriptive univariate statistics based on mean values, standard deviation and range for all parameters were determined for male and female populations (Callejón et al., 2017). The Student's t-test (P < 0.001) was used to test the equality of means for each variable. Statistical analysis was performed using Microsoft Excel 5.0 (Feliú et al., 2000). Thus, biometric characters of *Trichuris* sp. were compared between different hosts and the most significant parameters were assayed for a morphometric study (Table 1, Table 2).

**Table 1:** Biometric data of *Trichuris* adult male groups: *T. suis* isolated from *S. s. domestica*, *T. trichiura* isolated from *P. troglodytes*, *T. colobae* from *C. g*. *kikuyensis*, *T. ursinus* from *P. ursinus* and *Trichuris* sp. from *M. sylvanus*. † Significant differences between *T. suis* (Cutillas et al., 2009), *T. trichiura* (Cutillas et al., 2009), *T. colobae* (Cutillas et al., 2014) and *T. ursinus* (Callejón et al., 2017) compared to *Trichuris* sp. from *M. sylvanus* (P < 0.001).

	*T. trichiura* from *Pan troglodytes* (Cutillas et al., 2009)				*T. colobae* from *C. g. kikuyensis* Cutillas et al. (2014)				*Trichuris* sp. from *Macaca sylvanus* (Present study)				*Trichuris ursinus* from *Papio ursinus* (Callejón et al., 2017)				*T. suis* from *Sus scrofa domestica* (Cutillas et al., 2009)			
	MAX	MIN	Б	σ	MAX	MIN	Б	σ	MAX	MIN	Б	σ	MAX	MIN	Б	σ	MAX	MIN	Б	σ
M1	3.60	3.20	3.38	0.12	4.10	2.70	3.45	0.37	3.90	3.00	3.45	0.25	4.10	3.20	3.70	0.24	5.00	3.50	4.04 †	0.40
M2	2.30	1.80	2.08	0.13	3.50	2.10	2.64	0.37	2.80	1.90	2.19	0.27	2.70	2.10	2.43	0.19	3.30	2.30	2.61	0.29
LP	1.40	1.20	1.30	0.05	1.20	0.60	0.81 †	0.31	1.70	0.80	1.25	0.22	1.40	1.10	1.27	0.07	1.70	1.20	1.43	0.12
M3	0.31	0.09	0.14	0.07	0.10	0.07	0.08 †	0.01	0.18	0.12	0.14	0.02	0.20	0.15	0.17 †	0.01	0.23	0.16	0.20 †	0.02
M4	0.60	0.33	0.09 †	0.47	0.52	0.23	0.40 †	0.09	0.74	0.49	0.61	0.07	0.80	0.53	0.62	0.07	0.94	0.76	0.87 †	0.06
M5	0.32	0.15	0.22 †	0.05	0.26	0.13	0.20 †	0.04	0.49	0.25	0.37	0.06	0.46	0.30	0.38	0.03	0.40	0.28	0.35	0.04
M6	0.42	0.40	0.41 †	0.01	1.80	0.60	0.99 †	0.42	0.64	0.33	0.49	0.09	0.64	0.30	0.42	0.09	0.88	0.50	0.66	0.12
M7	0.88	0.59	0.67 †	0.09	3.95	2.33	3.36 †	0.44	1.57	1.10	1.33	0.14	2.29	1.10	1.71 †	0.35	1.76	1.00	1.43	0.27
M8	2.22	1.61	1.94 †	0.26	2.07	1.48	1.64 †	0.17	3.23	2.23	2.65	0.23	2.29	1.90	2.10 †	0.12	2.57	2.05	2.35 †	0.15
M9	0.22	0.16	0.21 †	0.02	0.65	0.26	0.49 †	0.09	1.23	0.53	0.93	0.20	0.69	0.23	0.54 †	0.14	0.76	0.16	0.39 †	0.19
M10	0.05	0.02	0.04 †	0.01	0.05	0.02	0.04 †	0.01	0.08	0.04	0.06	0.01	0.10	0.06	0.08 †	0.02	0.10	0.04	0.05	0.02
M11	0.06	0.03	0.04 †	0.01	0.06	0.03	0.04 †	0.01	0.08	0.04	0.06	0.01	0.10	0.05	0.09	0.14	0.08	0.06	0.07 †	0.01
M12	0.12	0.04	0.08	0.11	0.12	0.04	0.08	0.11	0.09	0.06	0.07	0.01	0.10	0.06	0.07	0.01	0.23	0.06	0.13 †	0.01
M13	2.98	1.32	2.14 †	0.47	2.98	1.32	2.14 †	0.47	5.19	2.90	4.06	0.58	2.34	1.55	1.84 †	0.43	2.34	1.99	2.15 †	0.14
M14	2.90	2.15	2.45 †	0.20	2.46	2.15	2.45	0.33	2.34	1.32	1.97	0.29	2.67	1.44	1.86	0.33	5.51	3.63	3.86 †	0.25
M15	3.29	2.2	2.87 †	0.28	3.06	2.55	2.87 †	0.28	2.75	1.55	2.20	0.36	3.79	2.77	3.18 †	0.27	4.92	3.42	4.31 †	0.54

M1 = Total body length of adult worm; M2 = Length of esophageal region of body; LP = Length of posterior region of body; M3 = Width of esophageal region of body; M4 = Maximum width of posterior region of body (thickness); M5 = Body width in the place of junction of esophagus and the intestine; M6 = Distance from the head end to beginning of bacillary stripes; M7 = Length of bacillary stripes; M8 = Length of spicule; M9 = Maximum length of spicule sheath; M10 = Width of proximal end of spicule; M11 = Width of spicule sheath at the tail end of body; M12 = Maximum width of spicule sheath; M13 = Distance between posterior part of testis and tail end of body; M14 = Length of ejaculatory duct; M15 = Length of distal cloacal tube. Б = Arithmetic mean. σ = Standard deviation.

**Table 2:** Biometric data of *Trichuris* adult female groups: *T. suis* isolated from *Sus scrofa domestica*, *T. trichiura* isolated from *P. troglodytes*, *T. colobae* from *C. g*. *kikuyensis*, *T. ursinus* from *P. ursinus* and *Trichuris* sp. from *Macaca sylvanus*. † Significant differences between *T. suis* (Cutillas et al., 2009), *T. trichiura* (Cutillas et al., 2009), *T. colobae* (Cutillas et al., 2014) and *T. ursinus* compared to *Trichuris* sp. from *M. sylvanus* (P < 0.001).

	*T. trichiura* from *Pan troglodytes* (Cutillas et al., 2009)				*T. colobae* from *C. g. kikuyensis*(Cutillas et al., 2014)				*Trichuris sp.* from *Macaca sylvanus* (Present study)				*Trichuris ursinus* from *Papio ursinus* (Callejón et al. (2017))				*T. suis* from *Sus scrofa* domestica (Cutillas et al., 2009)			
	MAX	MIN	Б	σ	MAX	MIN	Б	σ	MAX	MIN	Б	σ	MAX	MIN	Б	σ	MAX	MIN	Б	σ
F1	4.20	2.00	3.34	0.78	5.20	4.10	4.60 †	0.37	3.80	3.00	3.41	0.25	4.90	3.00	3.80	0.55	5.70	3.60	4.50 †	0.70
F2	3.30	1.30	2.53	0.68	3.80	3.00	3.34 †	0.31	2.60	1.80	2.19	0.23	3.70	2.00	2.60	0.50	4.10	2.70	3.30 †	0.41
LP	1.00	0.60	0.81 †	0.14	1.40	1.20	1.22	0.14	1.40	0.90	1.21	0.16	1.50	1.00	1.21	0.15	1.60	0.90	1.15	0.32
F3	0.19	0.01	0.11	0.05	0.15	0.08	0.11 †	0.02	0.18	0.13	0.15	0.01	0.19	0.15	0.17 †	0.02	0.24	0.16	0.20	0.06
F4	0.64	0.40	0.45 †	0.08	0.77	0.46	0.60 †	0.09	0.81	0.64	0.72	0.05	0.89	0.54	0.68	0.08	1.04	0.77	0.89 †	0.11
F5	0.23	0.13	0.17 †	0.03	0.31	0.16	0.23 †	0.04	0.48	0.36	0.42	0.03	0.45	0.35	0.40	0.02	0.46	0.24	0.32 †	0.08
F6	0.64	0.48	0.56	0.11	1.03	0.51	0.82 †	0.18	0.76	0.42	0.50	0.09	0.62	0.42	0.52	0.07	0.66	0.52	0.61 †	0.05
F7	0.95	0.36	0.65	0.30	4.92	2.90	3.50 †	0.79	1.71	0.90	1.44	0.21	2.00	1.29	1.70	0.23	1.92	1.45	1.69	0.19
F8	1.29	0.05	0.83	0.40	1.65	0.95	1.29 †	0.29	1.99	0.73	1.12	0.35	2.81	1.88	2.26 †	0.33	1.80	0.75	1.33	0.34
F9	0.11	0.03	0.05	0.03	0.08	0.05	0.07	0.01	0.09	0.02	0.05	0.02	0.12	0.06	0.08 †	0.02	1.00	0.60	0.80 †	0.14
F10	0.24	0.11	0.61	0.06	0.39	0.21	0.29	0.05	0.33	0.15	0.25	0.05	0.38	0.03	0.18	0.10	0.28	0.22	0.24	0.03
F11	0.22	0.20	0.21 †	0.01	0.45	0.20	0.32 †	0.08	0.84	0.40	0.61	0.14	0.75	0.10	0.47	0.22	0.65	0.48	0.60	0.05
F12	0.53	0.32	0.43	0.15	1.90	0.93	1.49 †	0.30	0.48	0.19	0.30	0.09	1.71	0.50	1.04 †	0.35	1.42	0.65	0.99 †	0.31
F13	0.67	0.65	0.66 †	0.01	1.20	0.50	0.79 †	0.27	0.14	0.05	0.11	0.04	1.32	0.77	0.99 †	0.20	1.20	0.72	0.92 †	0.18

F1 = Total body length of adult worm; F2 = Length of oesophageal region of body; LP = Length of posterior region of body; F3 = Width of esophageal region of body; F4 = Maximum width of posterior region of body (thickness); F5 = Body width in the place of junction of oesophagus and the intestine; F6 = Distance from the head end to beginning of bacillary stripes; F7 = Length of bacillary stripes; F8 = Length of vagina; F9 = Diameter of vulva turned over the surface of body; F10 = Distance of vulva from place of junction of oesophagus and the intestine; F11 = Distance of posterior loop of uterus from tail end of body; F12 = Distance of tail end of body and posterior fold of seminal receptacle; F13 = Length of muscular zone of the oesophagus. Б = Arithmetic mean. σ = Standard deviation.

Morphological variation is quantified by geometrical morphometrics (Rohlf and Marcus, 1993), a technique offering an estimate of size by which different axes of growth are integrated into a single variable (the ‘‘centroid size’’; Bookstein, 1989). The estimate of size is contained in a single variable reflecting variation in many directions, as many as there are landmarks under study, and shape is defined as their relative positions after correction for size, position and orientation. With these informative data, and the corresponding software freely available to conduct complex analyses, significant biological and epidemiological features can be quantified more accurately (Dujardin, 2008). Current statistical techniques in morphometrics make it possible to test the null hypothesis of conspecific populations being simply the allometric extension of each other, provided a common allometric trend is identifiable (Rohlf and Marcus, 1993).

Multivariate analyses were applied to calculate the phenotypic variations among whipworm adults, using size-free canonical discriminant analysis on the covariance of log-transformed measurements to assess phenotypic variations between the samples. These analyses are applied to exclude the effect of within-group ontogenetic variations by reducing the effect of each character on the first pooled within-group principal component (a multivariate size estimator) (Dos Reis et al., 1990). The principal component analysis (PCA) is used to summarize most of the variations in a multivariate dataset in a few dimensions (Dujardin and Le Pont, 2004). The resulting “allometry-free”, or size-free, variables were submitted to a canonical variate analysis (CVA), and Mahalanobis distances were derived (Mahalanobis, 1936). The Mahalanobis distance is a statistical technique that can be used to measure how distant a point is from the center of a multivariate normal distribution, i.e. in the present analysis, the degree of similarity between whipworm populations was assessed through pairwise Mahalanobis distances. Phenotypic analysis of whipworm adults was conducted using various modules of the CLIC package version 97 (Dujardin and Slice, 2007; Dujardin et al., 2010), which is freely available at http://mome-clic.com, and BAC v.2 software (Dujardin, 2002; Valero et al., 2009), both used for multivariate analyses of the morphometric data. Furthermore, Mahalanobis distances were calculated using CLIC software and tested by nonparametric permutation tests with 1,000 iterations each.

The results were statistically significant when P < 0.05. The following non-redundant measurements (one measurement is not included in another) used for whipworm adults were: M4, M8 and M9 for males; ratio: F2/LP, F4 and F9 for females.

## 3 Results

The Student's T test showed a number of measurements to be significant for subsequent morphometric analyses: maximum width of posterior region of the body (thickness) (M4), length of spicule (M8), maximum length of spicule sheath (M9), distance between posterior part of testis and tail end of body (M13) and length of distal cloacal tube (M15) in males, and maximum width of posterior region of body (thickness) (F4) and length of the muscular zone of the oesophagus (F13) in females. All these measurements are presented in millimeters (Table 1, Table 2).

**Fig. 1:**
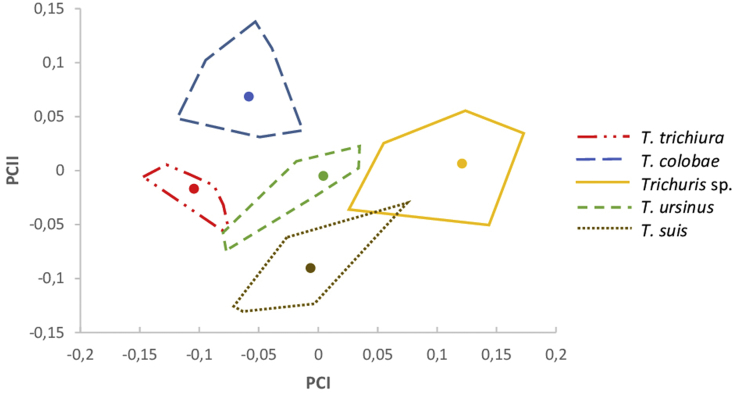
Factor map corresponding to adult *Trichuris* sp. males derived from different host primate species (*Macaca sylvanus*, *Pan troglodytes*, *Colobus guereza kikuyensis*, *Papio ursinus*) and the pig (*Sus scrofa domestica*) from zoos and abattoirs, respectively, in Spain. Samples are projected onto the first (PC1, 64%) and second (PC2, 29%) principal components. Each group is represented by its perimeter. Circles represent the centroid in each community.

**Fig. 2:**
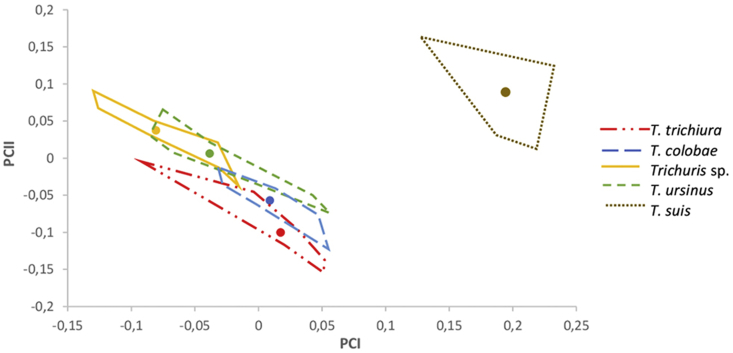
Factor map corresponding to adult *Trichuris* sp. females derived from four different host primate species (*Macaca sylvanus*, *Pan troglodytes*, *Colobus guereza kikuyensis*, *Papio ursinus*) and the pig (*Sus scrofa domestica*) from zoos and abattoirs, respectively, in Spain. Samples are projected onto the first (PC1, 61%) and second (PC2, 35%) principal components. Each group is represented by its perimeter. Circles represent the centroid in each community.

The study of the influence of the host species on the adult size was carried out by PCA in *Trichuris* spp. from *M. sylvanus*, *P. troglodytes*, *C. g. kikuyensis*, *P. ursinus* and *S. s. domestica*. Different parameters were included according to the significant value obtained by the Student's T test. Therefore, three measurements for each population: M4, M8 and M9 for males, and F2/LP, F4 and F9 for females were used. *Trichuris* spp. variables from the primates and pigs all correlated significantly with PC1, contributing 64% to the overall variation in males and 61% in females. The resulting factor maps for male and female populations of *Trichuris* sp. adults are represented in Fig. 1 and Fig. 2, respectively.

On the one hand, the first factor map clearly illustrates the global size differences in the male *Trichuris* sp. populations analyzed, showing a larger size in males collected from macaques (Fig. 1). All of the adult male communities are well grouped in the factor map, with a lack of overlapping areas between them. Therefore, each population appeared separate from each other. Only parasites from macaques and pigs showed a partial overlap but with no inconvenience in the identification of the communities. Furthermore, the CS differences in males between three groups (*T. trichiura* vs *Trichuris* sp, *T. trichiura* vs *T. colobae* and *Trichuris* sp vs *T. colobae*) presented the highest values.

On the other hand, the female factor map does not show global size differences in four of the five *Trichuris* sp. populations. Thus, two zones could be distinguished: one zone was made up of the four primate communities, while the other zone consists only of pigs. The primates show a wide overlap area that does not allow a clear identification of each adult female population, although the CS differences in females between three groups (*T. trichiura* vs *Trichuris* sp, *T. trichiura* vs *T. ursinus* and *Trichuris* sp vs *T. colobae*) presented clear differences. Therefore, the exception are adult *Trichuris* sp. females collected from pigs making up an independent group from the primate communities and with no overlap between them, only a bigger size in female adults retrieved from pigs being patent (Fig. 2).

The degree of similarity between whipworm populations was assessed through pairwise Mahalanobis distances. These distances were calculated comparing males with each other (Table 3), respectively, comparing females with each other (Table 4). When comparing males of *Trichuris* species from NHPs vs *Trichuris* species from pigs, larger distances were detected than in the inter-NHPs comparison. Similarly, when comparing females of *Trichuris* species from NHPs vs *Trichuris* species from pigs, larger distances were detected than in the inter-NHPs comparison. In the inter-NHPs comparison in males of *Trichuris* larger distances were detected *T. trichiura* vs *Trichuris* sp and *T. trichiura* vs *T. colobae* were compared. Similarly, in the inter-NHPs comparison in females of *Trichuris*, larger distances were detected *T. trichiura* vs *Trichuris* sp, *T. trichiura* vs *T. ursinus* and *Trichuris* sp vs *T. colobae* were compared. In general, larger distances between females than between males were detected when Trichuris species from NHPs vs Trichuris species from pigs where compared, revealing that the phenotype of T. suis vs NHP whipworm species is more divergent in females than in males. These results agree with the above-mentioned analysis obtained in Fig. 1, Fig. 2.

**Table 3:** Mahalanobis distances between *Trichuris* adult male groups: *T. suis* isolated from *S. s. domestica*, *T. trichiura* isolated from *P. troglodytes*, *T. colobae* from *C. g. kikuyensis*, *T. ursinus* from *P. ursinus* and *Trichuris sp.* from *M. sylvanus*.

	*T. trichiura*	*T. colobae*	*Trichuris* sp.	*T. ursinus*	*T. suis*
*T. trichiura*	0.00				
*T. colobae*	3.05	0.00			
*Trichuris* sp.	3.53	2.34	0.00		
*T. ursinus*	1.82	1.24	2.58	0.00	
*T. suis*	1.56	3.58	4.80	2.46	0.00

**Table 4:** Mahalanobis distances between *Trichuris* adult female groups: *T. suis* isolated from *S. s. domestica*, *T. trichiura* isolated from *P. troglodytes*, *T. colobae* from *C. g. kikuyensis*, *T. ursinus* from *P. ursinus* and *Trichuris sp.* from *M. sylvanus*.

	*T. trichiura*	*T. colobae*	*Trichuris* sp.	*T. ursinus*	*T. suis*
*T. trichiura*	0.00				
*T. colobae*	2.10	0.00			
*Trichuris* sp.	5.10	3.10	0.00		
*T. ursinus*	3.73	1.79	1.38	0.00	
*T. suis*	6.01	7.24	8.77	7.73	0.00

## 4 Discussion

The systematics of the genus *Trichuris* Roederer, 1761 is controversial at species level. Different authors have cited synonymies (Oliveros et al., 2000), cryptic species (Callejón et al., 2012) or new species (Cutillas et al., 2014; Robles et al., 2014; Callejón et al., 2017). Many of these studies have been based on morphometric and molecular data since Dujardin (1845) reviewed the genus for the first time.

Furthermore, we have carried out the first morphometric study applied to adult *Trichuris* species derived from different host primates and the pig, in order to explore complementary methods of morphological phenotypic characterization. Previous studies demonstrated the difficulty in discriminating populations of the genus *Trichuris* from primates (Cutillas et al., 2009, 2014; Callejón et al., 2017) as it usually contains cryptic species. In view of these problems, an additional method was required to elucidate different species. The present study demonstrates that morphometrics is a useful tool to shed light on this topic.

As for adult male *Trichuris*, we achieved the differentiation of all of the communities, obtaining well defined different areas for each population. These results are in agreement with the molecular biology analysis applied to the adult male samples that allow the identification of different species of *Trichuris* (Cutillas et al., 2009, 2014; Callejón et al., 2017). The results confirm that each community of male *Trichuris* sp. adults possesses its own morphological identity.

The size of female *Trichuris* populations does not follow a host-dependent pattern, probably due to the absence of representative measurements. Only the females retrieved from pigs presented a larger size than the females collected from primates (Fig. 2). All combinations of measurements considered always led to similar factor maps, with wide overlap areas between them (data not shown). These results agree with those reported previously (Cutillas et al., 2009, 2014; Callejón et al., 2017), in which the differentiation of female *Trichuris* was not achieved. Females of trichurids are more difficult to differentiate than males, and some authors suggested that the structure of the vulva could be used for species differentiation (Chandler, 1930; Ooi et al., 1993; Barus et al., 1978; Rickard and Bishop, 1991; Gibbons, 1986). Tenora et al. (1993) stated that there are two different types of vulva in trichurid females: with and without spines. *T. suis* belongs to the group of species possessing the vulvar region equipped with cuticle spines present of various forms and different numbers. Furthermore, Spakulová (1994) found that the combination of seven metric characters distinguished the whipworms from pigs from those of humans.

According to different reports mentioned above, the main gaps in the systematics of the genus *Trichuris* are:

1. The absence of comparative morpho-biometric data using multiple parameters, statistical tests (e.g. the Student's Test, P < 0.001) and geometrical morphometrics applied to the taxonomic study of different species of *Trichuris.*
2. The reports about different genetic lineages in *T. trichiura* and species of this genus parasitizing NHPs are not supported by morphobiometric studies. Thus, future research priorities should include morphological, morphometric and molecular analyses of individuals of *Trichuris* sp., in order to compare data.

These issues can be addressed by means of:

1. A classical taxonomic study based on the analysis of 15–20 biometric parameters and statistical tests, along with geometrical morphometrics based on Principal Component analysis of different species of *Trichuris* isolated from different hosts and geographic areas involving the four continents: Europe, Africa, America and Asia.
2. The amplification and sequencing of nuclear and mitochondrial genes (genome) of these *Trichuris* species.
3. The phylogenetic and phylogeographic study based on whole-genome and through analysis of concatenated sequences obtained from these species from different geographical localities, to shed light on the degree of divergence with respect to *T. trichiura.*
4. The comparative study of sequences to obtain new pharmacological targets in therapy for autoimmune diseases such as Crohn's disease, as well as new treatments of the parasitism by these species in livestock.

## 5 Conclusions

The results obtained revealed strong support for geometrical morphometrics as a useful tool to differentiate male *Trichuris* populations. Therefore, morphometrics in combination with other techniques, such as molecular biology analyses, ought to be applied to further the differentiation of male populations.

On the other hand, morphometrics applied to female *Trichuris* species does not seem to contribute new information as all the measurement combinations of obtained from females always showed similar results. The application of other techniques to differentiate populations is warranted given the difficulty in finding representative measurements in females.

Combining genetic and morphometric analyses seem to be the key factor to enable the differentiation of *Trichuris* sp. in the near future.

## Formatting of funding sources

This *research* has be en funded by a grant from the Ministry of Economy, Industry and Competitiveness (CGL2017-83057), which included FEDER funds, the Junta de Andalucía (BIO-338) and a grant from the V Plan Propio de Investigación of the University of Seville, Spain.

## Conflicts of interest

The authors declare no conflicts of interest.
